# Transdermal iontophoresis delivery system for terazosin hydrochloride: an *in vitro* and *in vivo* study

**DOI:** 10.1080/10717544.2021.1889719

**Published:** 2021-02-23

**Authors:** Changzhao Jiang, Xiumei Jiang, Xiumin Wang, Jiaxu Shen, Mengjie Zhang, Leilei Jiang, Rui Ma, Tingting Gan, Yingbiao Gong, Jincui Ye, Wenyan Gao

**Affiliations:** a Key Laboratory of Neuropsychiatric Drug Research of Zhejiang Province, Institute of Materia Medica, Hangzhou Medical College, Hangzhou, China; b Collaborative Innovation Center of Green Pharmaceuticals, Zhejiang University of Technology, Hangzhou, China

**Keywords:** Terazosin hydrochloride, iontophoresis, transdermal drug delivery, pharmacokinetics, blood pressure

## Abstract

This study aimed to construct a transdermal iontophoresis delivery system for terazosin hydrochloride (IDDS-TEH), which included a positive and negative electrode hydrogel prescription. Intact guinea pig skin was used as a model for the skin barrier function, and the current intensity, terazosin hydrochloride (TEH) concentration, pH, competitive salt, and transdermal enhancer properties were studied. The blood drug concentration was determined in Sprague–Dawley (SD) rats using HPLC, and the antihypertensive effects of IDDS-TEH were evaluated in spontaneously hypertensive rats (SHRs). The results showed that the steady-state penetration rate of TEH increased (from 80.36 µg·cm^−2^·h^−1^ to 304.93 µg·cm^−2^·h^−1^), followed by an increase in the current intensity (from 0.10 mA·cm^−2^ to 0.49 mA·cm^−2^). The pH values also had a significant influence on percutaneous penetration. The blood concentration of IDDS-TEH was significantly higher (*p* < .05) than with passive diffusion, which could not be detected. The main pharmacokinetic parameters of the high current group (0.17 mA·cm^−2^) and the low current group (0.09 mA·cm^−2^) were AUC_0–_*_t_*: 5873.0 ng·mL^−1^·h and 2493.7 ng·mL^−1^·h, respectively. Meanwhile, the pharmacodynamic results showed that IDDS-TEH significantly decreased the blood pressure of SHRs compared with the TEH hydrogel without loading current. Therefore, TEH could be successfully delivered by the transdermal iontophoresis system *in vitro* and *in vivo*, and further clinical studies should be explored to develop a therapeutically useful protocol.

## Introduction

Terazosin, a selective, quinazoline-derived post-synaptic alpha-1 antagonist, was approved by the Food and Drug Administration (FDA) in 1987 as a treatment for hypertension and then approved in 1993 as a treatment for lower urinary tract symptoms associated with benign prostatic hyperplasia (Piascik & Perez, 2001; Yang & Raja, 2020). Terazosin is commonly available in oral capsule formulations as an HCl salt with 1, 2, 5, or 10 mg formulations. However, there are many adverse effects of terazosin due to large fluctuation of plasma concentration by gastrointestinal track administration (Oestreich et al., 2020; Hundemer et al., 2021), for example, severe dizziness, weakness, and orthostatic hypotension. Orthostatic vital signs are obtained after the first dose to exclude postural hypotension. If used for hypertension, orthostatic blood pressures may be checked regularly during the titration interval to confirm efficacy (Titmarsh & Monk, 1987). Hence, an alternative delivery route needs to be explored.

Transdermal iontophoresis is a physically noninvasive method that involves applying a low electrical potential gradient across the skin to enhance molecular transport and is widely used for transdermal drug delivery (Kanikkannan, 2002; Mohammed et al., 2016). The iontophoresis system consists of a power supply, electrode, control circuit, drug storehouse, and electrolyte storehouse (Kanikkannan, 2002). Iontophoresis equipment primarily measures the electric field force, electroosmotic flow, or electric skin structure changes. In addition, the mechanism of reverse iontophoresis is simple; like charges repel and opposite charges attract (Byrne et al., 2018). The transdermal drug delivery system (TDDS) is one potential route for the systemic delivery of drugs that allows drugs to be administered in an individual-dose regimen and provides prolonged treatment (Shelke et al., 2007). To become a feasible candidate for TDDS (Wiedersberg & Guy, 2014), modest molecular weight (MW; 400–500 Da), balanced lipophilicity (log(octanol–water partition coefficient), log P, ideally around 2 to 3), and a measurable solubility both in oil and in water were required. Commonly, TEH could not be passive into the blood by percutaneous absorption, whereas the transdermal iontophoresis system improved the absorption of TEH in a grade extent. Compared with TDDS, transdermal iontophoresis, based on the original passive transport, can deliver drugs into the skin over a shorter time. It is particularly suitable for delivering ionic and small peptide drugs (Bhattaccharjee et al., 2020). In recent years, iontophoresis has been widely used in drug delivery for the treatment of local anesthesia, analgesia, hyperhidrosis, psoriasis, and skin cancer (Wan et al., 2018; Wanasathop & Li, 2018; Murota et al., 2020; Yamada & Prow, 2020; Yamaga et al., 2020). The portable iontophoresis drug-containing products lidosite, ionsys, and zecuity have been approved by the FDA (Panchagnula et al., 2000). However, there are no studies regarding the terazosin hydrochloride iontophoresis system. In this study, terazosin hydrochloride was used as a model drug for preparing the iontophoresis drug delivery system, which may further expand the scope of indications for this drug.

An electroosmotic transdermal drug delivery system has the advantages of fewer adverse reactions, stable blood drug concentrations, and adjustable drug dosage according to the individual treatment regimens and the disease phase. Meanwhile, iontophoresis for the transdermal delivery of terazosin can monitor adverse reactions at any time, which can be halted upon adverse reactions induced by the drug. By loading a small current, the TEH blood concentration can reach the therapeutic dose, which is useful for clinical studies. It can also provide ideas for the secondary development of existing drugs for novel uses and expand the scope of application for transdermal drug delivery preparations. Moreover, the IDDS-TEH could probably be combined with wrist-type blood pressure monitoring equipment (Melville et al., 2018) in the future, which may make it succeed in refined administration through the feedback of blood pressure value.

Therefore, this work focused on evaluating the potential of iontophoresis in transdermal delivery of terazosin and formulating a new delivery system for terazosin capable of providing sustained and controlled release. Firstly, a kind of TEH hydrogel was formulated and optimized by central composite design-response surface methodology. Then the effects of five factors (i.e. current intensity, drug concentration, pH value, NaCl concentration, chemical penetration enhancer) on the penetration rate of IDDS-TEH were studied *in vitro* to understand the drug release ability of the IDDS-TEH. To investigate the effect of electroosmosis on TEH transdermal delivery *in vivo*, we also determined the pharmacokinetic characteristics using the SD rat model. And the antihypertensive pharmacological effects of IDDS-TEH were evaluated in spontaneously hypertensive rats (SHRs) model to demonstrate the therapeutic efficacy of IDDS-TEH.

## Materials and methods

### Materials

TEH was purchased from Weihai Disu Pharmaceutical Co. Ltd. (Weihai, China), and sodium hydroxypropyl methylcellulose (HPMC) was purchased from Anhui Sunhere Pharmaceutical Excipients Co., Ltd. (Huainan, China). All other reagents and solvents were of analytical grade or HPLC grade purchased from local supplier. Guinea pigs originated from Wuxi Hengtai Experimental Animal Breeding Co. Ltd. (Wuxi, China), production license No: SCXK-(Su) 2015–0004. SD rats originated from Zhejiang Province Laboratory Animal Center (Hangzhou, China), production license No: SCXK-(Zhe) 2014–0001. Spontaneously hypertensive rats (SHRs) were obtained from Charles River Laboratories (Beijing, China), production license No: SCXK-(Jing) 2016–0006.

### Methods

#### Preparation of hydrogel

**Figure 1.**
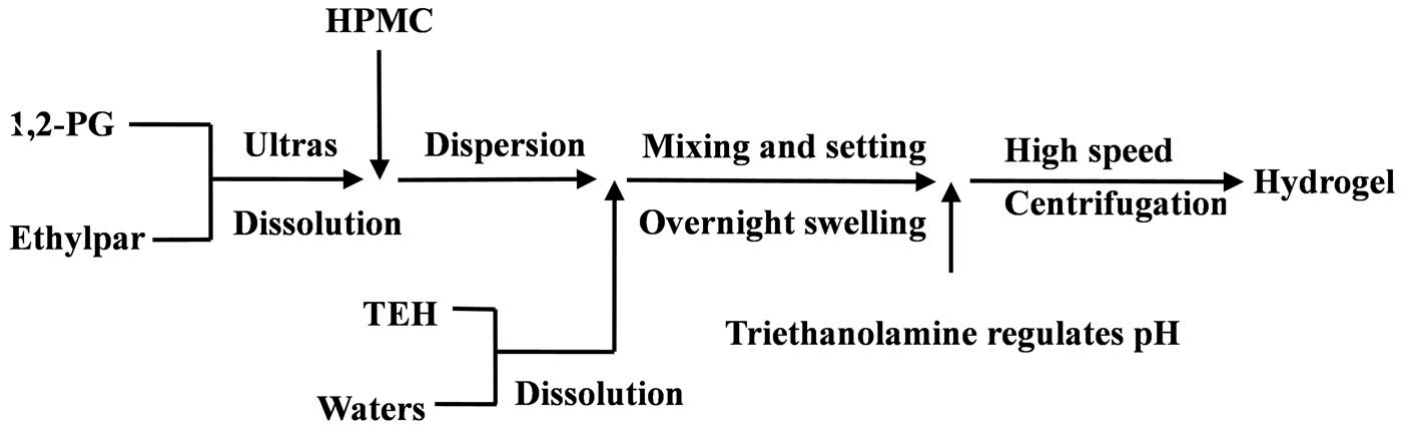
Diagram of TEH hydrogel preparation.

The preparation of the TEH hydrogel is shown in Figure 1. HPMC and 1,2-PG were dissolved and gradually mixed with water to avoid caking and insufficient swelling. The composition and dosage of TEH hydrogel for drug storage are listed in Table 1. The electrolytic hydrogel connected to the negative pole without drug and riethanolamine, which contained 0.2% phosphate buffer (pH = 7.4). The TEH hydrogel was optimized using the star point design-response surface method, shown in Supplementary File 1.

**Table 1.** Composition of TEH hydrogel formulae.

Formulation	Application	Usage (%)
TEH	Drug	0.10 − 1.00
HPMC	Excipient	2.18
1,2-PG	Humectant	14.60
Ethylparaben	Preservative	0.10
Triethanolamine	pH regulator	pH adjust to 4.8
Waters	Solution	-

#### Separation of guinea pig skin

The guinea pigs were stored at room temperature (18–24 °C) and 60 ± 10% humidity, with a light-dark cycle (12 h–12 h) for 7 days. After anesthetization with 4% chloral hydrate, the guinea pigs were shaved using an electric razor while taking care to avoid skin damage. The muscle and fat were carefully cleared using eye scissors, washed, and the hair and residual tissue on the skin surface was removed with normal saline. The skin was dried with filter paper, cut into 1.5 cm × 1.5 cm squares, placed in an aluminum-plastic bag, sealed, and stored at −20 °C.

#### In vitro skin iontophoresis study

Frozen guinea pigs were placed in normal saline at 32 °C, and the water was absorbed using filter paper. The skin was fixed on a diffusion cell. TEH hydrogel (1.5 g) was used for the diffusion cell, and 5.5 mL of phosphate buffer was added to the diffusion cell. The positive pole was connected to the hydrogel, and the negative pole was connected to the phosphate buffer. The electroosmosis equipment was turned on and maintained at a constant current intensity of 0–0.3 mA·cm^−2^ at a temperature of 32 °C. Receiving solution (1.0 mL) was collected at 1, 2, 4, 6, 8, and 10 h, and supplemented with the same volume of blank receiving solution. The permeation of the receiving solution was performed using a 0.22 μm needle filter and then analyzed using HPLC. The permeation parameters, namely accumulative amount (*Q*), steady-state flux (*J_ss_*), and *J_s_* enhancement ratio, were evaluated.

$$Q_{n}={(C}_{n}\text{ × }V_{0}\;+\;\sum_{I=1}^{n-1}C_{i}\text{ × }V)/A\;$$

*Note*: *Q*: per unit area of cumulative penetration after several hours; *C*: drug concentration; *V*: volume of the receiver solution at each time; *A*: effective penetration area. In this experiment, *V_0_* = 5.5 mL, *A* = 0.71 cm^2^, *V* = 1.0 mL.

According to the calculation of the accumulative amount, the *Q*–*t* curve was drawn, and the slope value of the curve was the permeability rate *J_ss_*.

$$J_{\mathrm{ss}}=\frac{dQ}{\text{dt}}$$

*Note*: ER is the ratio of *J_SS_* with and without penetration enhancer.

### Effect of current intensity

Guinea pig skin with 0.5% TEH hydrogel was divided into six groups (The intensity of iontophoresis equipment was adjusted and maintained at 0, 0.10, 0.20, 0.30, 0.39, and 0.49 mA·cm^−2^, respectively). Samples were collected at 1, 2, 4, 6, 8, and 10 h after administration. According to the TEH concentration analyzed by liquid chromatography at different time points, the cumulative penetration per unit area (*Q_n_*) at each time point of TEH under different intensities was calculated.

### Effect of drug concentration

The delivery of terazosin hydrochloride was adjusted to 0.1%, 0.3%, 0.5%, 0.7%, and 0.9% of the total TEH hydrogel. The output current intensity of the ion osmosis equipment was adjusted and maintained at a value of 0.49 mA·cm^−2^. Samples were collected at 1, 2, 4, 6, 8, and 10 h after administration, and the *Q_n_* of TEH at each time point was calculated.

### Effect of pH

The stored drug gel’s pH was adjusted to 4.81, 6.68, 7.40, and 8.10 using triethanolamine. A hydrogel pH of 4.81 was the initial pH value without triethanolamine in the formulation.

### Effect of NaCl concentration

The hydrogels were prepared with NaCl concentrations of 0.1, 0.3, 0.5, and 1.0%. The transdermal penetration test was performed using the same method described above.

### Effect of the chemical penetration enhancer

The optimized hydrogels were prepared with 10% ethanol, 5% dipentene, and 5% menthol. The transdermal penetration test was performed using the same method described above.

### In-vivo pharmacokinetics of IDDS-TEH in rats

Eighteen SD rats were randomly divided into three groups (current intensity was 0 mA·cm^−2^, 0.09 mA·cm^−2^, and 0.17 mA·cm^−2^ (when the current was higher than 0.17 mA·cm^−2^, the rats were restless and vocalized)). The number of males and females was balanced in each group. The rats were rapidly anesthetized by ether inhalation, and their back and abdominal hair were carefully removed. The rats were placed in a stainless-steel fixator, and the hydrogel (7.5 mg TEH in 1.5 g hydrogel) was connected to the positive pole on the back and the electrolyte hydrogel was connected to the negative pole on the abdomen. The current intensity was adjusted to an appropriate extent (0, 0.09, 0.17 mA·cm^−2^, respectively). Subsequently, the electroosmotic hydrogel and electrode poles with the remaining hydrogel on the skin were removed. Blood samples were collected at 0.5, 1, 2, 4, 8, 10, 12, 16, 24, and 34 h, centrifuged, and the plasma stored at −20 °C. It should be noted that the current was off at 10 h. The TEH in the plasma was measured using HPLC. The drug concentration was calculated using the pharmacokinetic analysis software DAS VER 2.0 (Mathematical Pharmacology Professional Committee of China, Shanghai, China).

### HPLC analysis of TEH in rat plasma

An accurately measured 200 μL internal standard (doxazosin mesylate) solution was added to a 2.0 mL centrifuge tube with 200 μL plasma, 50 μL NaOH (1.0 mol·L^−1^), and 1 mL methyl tert-butyl ether, mixed, and centrifuged at the force approximately 8,000 *g* for 10 min. Absorbed the supernatant to centrifuge tube, and the supernatant was then dried, the residues dissolved in 150 μL of the mobile phase, centrifuged at the force approximately 8,000 *g* for 15 min, and the supernatant collected for HPLC analysis. The establishment and validation of the HPLC analytical method for blood drug concentration were shown in Supplementary File 2.

### In-vivo pharmacodynamics of IDDS-TEH in SHRs

The SHRs were divided into two groups: the control group (7.5 mg/1.5 g TEH hydrogel on the skin without current intensity) and the experimental group (7.5 mg/1.5 g TEH hydrogel on the skin with a current intensity of 0.17 mA·cm^−2^). The SHRs were rapidly anesthetized by ether inhalation, and their back and abdominal hair were carefully removed. The TEH hydrogel was administered to the skin with or without current. Blood pressure measurements were performed using a noninvasive tail artery measurement. The rats were fixed and preheated for 20 min, and then the pressure signal was calibrated. The rat tail was through the compression sleeve, which was placed close to the tail root. The electroosmotic hydrogel and electrode poles were removed, and the remaining hydrogel on the skin of all SHRs was cleared after 10 h. Blood pressure, including systolic blood pressure (SBP) and diastolic blood pressure (DBP), was measured at 0 h (before administration), and 2, 4, 6, 8, 12, 24, and 32 h after administration of the TEH hydrogel.

### Statistical analysis

The data are expressed as the mean ± SD. The *t*-test was used to analyze the data. *p* < .05 was considered statistically significant.

## Results

### Effect of current intensity

**Figure 2.**
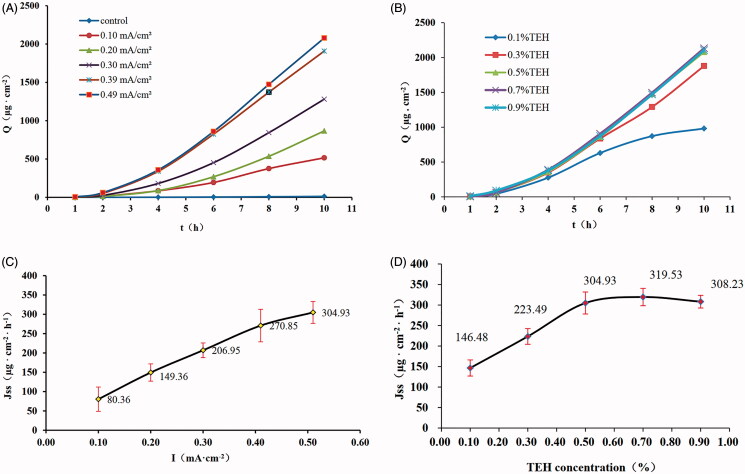
Effect of current intensity and drug concentrations on the penetration of IDDS-TEH. (A) Permeation kinetics curves for TEH at different current intensities. (B) Permeation kinetics curve of TEH at different concentrations. (C) relationship curve of *J_ss_*-current intensity (*n* = 4). (D) Relationship curve of *J_ss_*-TEH concentration (*n* = 4).

According to the *in vitro* guinea pig skin iontophoresis permeability test, the cumulative permeability (*Q*) with different current intensities was obtained, and quantitative analysis was performed with the TEH time–*Q* curve, as shown in Figure 2(A). The relationship curve of the transdermal flux (*J_ss_*)-current intensity (*I*) is shown in Figure 2(C). The results showed that the *J_ss_* increased gradually with increasing current intensity. The *J_ss_* was very close to zero without an electric current (control). When the current intensity was gradually increased from 0.10 mA cm^−2^ to 0.49 mA·cm^−2^, the *J_ss_* increased from 80.36 µg cm^−2^ h^−1^ to 304.93 µg cm^−2^·h^−1^. The linearity was significant, with a regression coefficient (*R*^2^) of 0.986.

### Effect of drug concentration

Quantitative analysis was performed using the concentration-*J_ss_* curve of TEH at different concentrations, as shown in Figure 2(B,D). The TEH concentration and *J_ss_* were in a nonlinear relationship. When the drug concentration was less than 0.5%, *J_ss_* increased with TEH concentration. However, when the concentration of TEH was more than 0.5%, the increase in *J_ss_* was limited. In addition, there was no significant difference (*p* > .05) in *J_ss_* between the TEH at 0.5, 0.7, and 0.9%.

The transdermal iontophoresis flux of some drugs had a relatively linear dependence on their supply chamber concentrations. However, with an increasing drug concentration, the increasing *J_ss_* of drugs was limited (Marro et al., 2001). Thus, the number of skin permeation channels may limit drug penetration.

### Effect of pH

**Figure 3.**
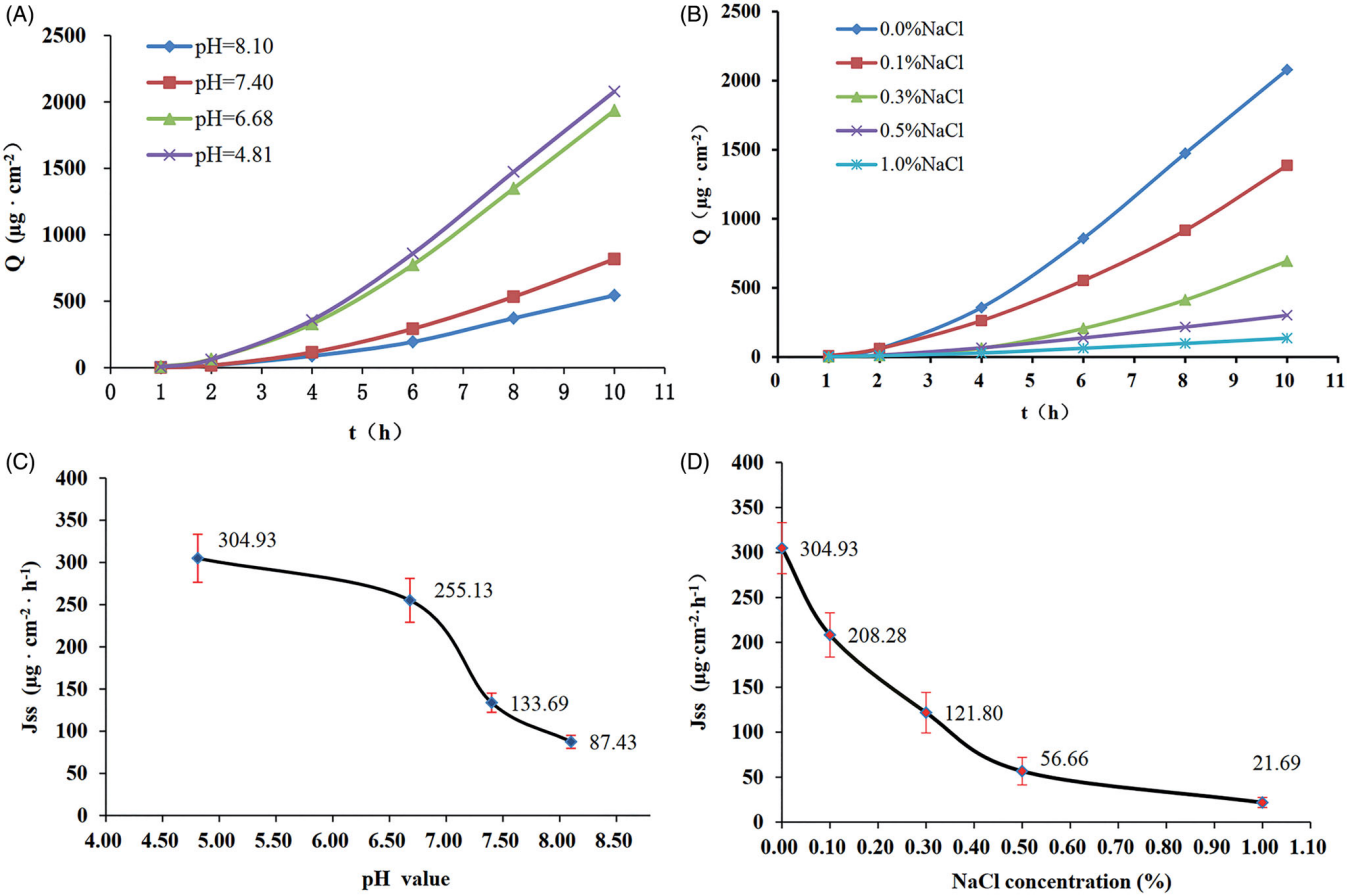
Effect of pH and NaCl concentrations on the penetration of IDDS-TEH. (A) Permeability kinetics curves for TEH under different pH conditions.(B) Permeation kinetics curves at different NaCl concentration. (C) *J_ss_*-pH value relationship curve (*n* = 4). (D) Relationship curve of steady transdermal penetration rate-NaCl concentration.

The *Q*–*t* and *J_ss_*-pH curves of TEH at different pH values are shown in Figure 3(A,C). The results suggested that the pH of the drug during storage significantly affected transdermal iontophoresis of TEH. The *J_ss_* of TEH decreased as the pH of the hydrogel increased from 4.81 to 8.10. ER was 0.84-, 0.44-, and 0.29-fold lower at pH 6.68, 7.40, and 8.10, respectively, than at pH 4.81.

### Effect of NaCl concentration

The *Q*–*t* and *J_ss_*-concentration curves of TEH with different NaCl concentrations are shown in Figure 3(B,D). The results showed that NaCl attenuated THE’s transdermal iontophoresis. *J_ss_* decreased significantly with increasing NaCl concentration in the hydrogel formulation. ER was 0.68, 0.40, 0.19, and 0.07 when NaCl concentration was 0.1, 0.3, 0.5, and 1.0%, respectively.

### Effect of chemical penetration enhancer

**Figure 4.**
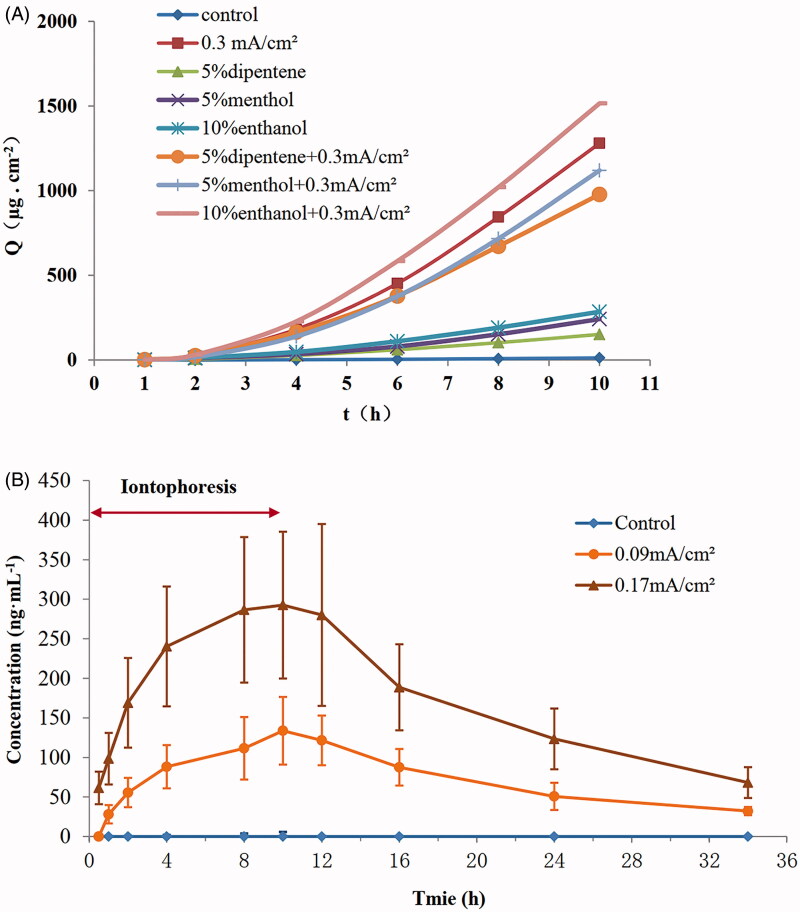
Effect of chemical penetration enhancers on the penetration of IDDS-TEH and pharmacokinetics of IDDS-TEH in rats. (A) Permeation kinetic curves of TEH under different chemical penetration enhancers (*n* = 4). (B) TEH blood concentration-time curve (*n* = 6).

The *Q*–*t* curve of TEH with different chemical penetration enhancers is shown in Figure 4(A), and the *J_ss_* and enhancement ratio (ER) is shown in Table 2. The results showed that 5% dipentene and 5% menthol inhibited the transdermal iontophoresis of TEH. Moreover, 10% ethanol combined with 0.3 mA cm^−2^ slightly increased the ER. The combination with electroosmotic gel had no apparent effect on promoting the transdermal delivery of terazosin hydrochloride.

**Table 2.** Permeation kinetic parameters of TEH under iontophoresis with different chemicals.

Groups	*J_ss_* (µg·cm^−^²·h^−1^)	ER
Control	2.05 ± 0.96	1
0.30 mA·cm^−2^	206.95 ± 18.96	100.95
5% dipentene	22.63 ± 8.65	11.04
5% dipentene + 0.30 mA·cm^−2^	149.93 ± 25.32	73.14
5% menthol	40.40 ± 12.83	19.71
5% menthol + 0.30 mA·cm^−2^	185.90 ± 31.34	90.68
10% enthanol	43.60 ± 9.78	21.27
10% enthanol + 0.30 mA·cm^−2^	232.83 ± 32.01	113.57

### Pharmacokinetics of IDDS-TEH in rats

As shown in Figure 4(B) and Table 3, HPLC detected no TEH when there was no current intensity. However, the TEH concentration of blood was significantly increased when the current intensity was 0.09 and 0.17 mA·cm^−2^. The AUC_(0–_*_t_*_)_, *t*_1/2_(*β*), *T_max_*_,_ and *C_max_* for 0.09 mA·cm^−2^ were 2493.7 ng·mL^−1^·h, 10.1h, 10 h, and 135.3 ng·mL^−1^. The AUC_(0–_*_t_*_)_, *t*_1/2_(*β*), *T_max_*_,_ and *C_max_* for 0.17 mA·cm^−2^ were 5873.0 ng·mL^−1^·h, 11.4h, 10 h, and 292.6 ng·mL^−1^.

**Table 3.** The main pharmacokinetic parameters of TEH percutaneous iontophoresis.

Parameters	Unit	Iontophoresis (0.09 mA·cm^−2^)	Iontophoresis (0.17 mA·cm^−2^)
AUC_(0–_*_t_*_)_	ng·mL^−1^·h	2493.7	5873.0
AUC_(0–∞)_	ng·mL^−1^·h	2867.2	6990.5
*t*_1/2_(*β*)	h	10.1	11.4
*T_max_*	h	10	10
*C_max_*	ng·mL^−1^	135.3	292.6

### Pharmacodynamics of IDDS-TEH in SHRs

**Figure 5.**
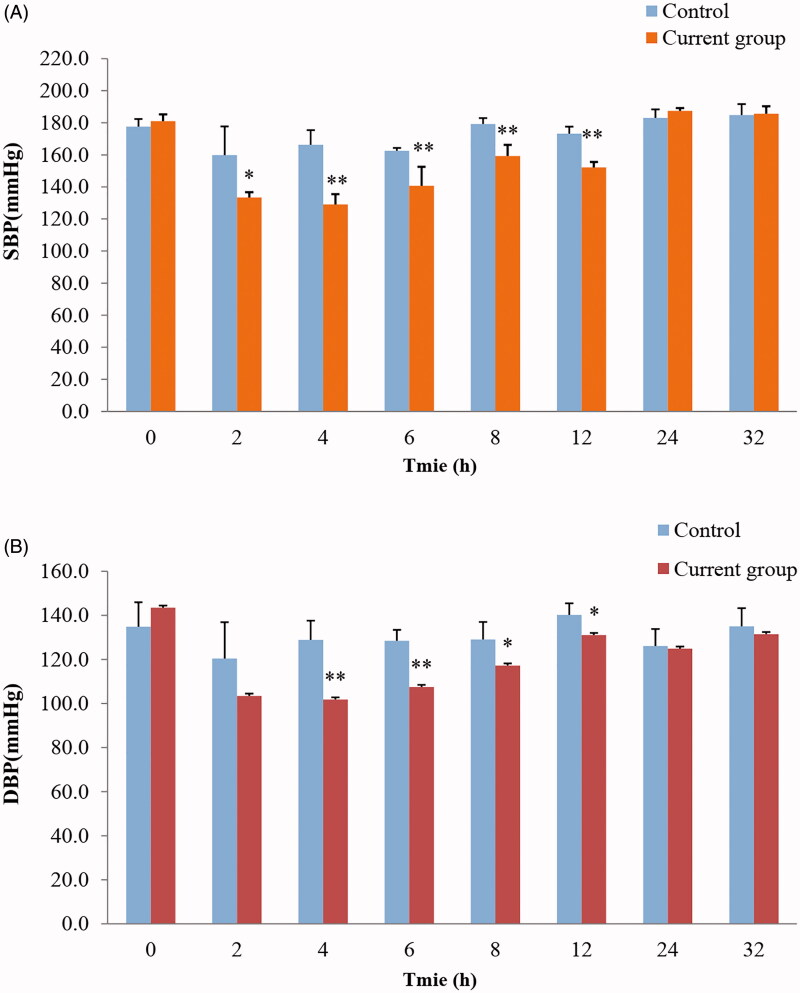
Effect of IDDS-TEH on blood pressure of SHRs under identical experimental conditions. Data are presented as the mean ± S.D. (*n* = 5). ‘*’ Indicates a significant difference (*p* < .05) and ‘**’ indicates a significant difference (*p* < .01).

TEH has a significant hypotensive effect on SHRs (da Silva et al., 2008; Aa et al., 2010). After transdermal administration of TEH hydrogel to SHRs with appropriate loading current, the effect of IDDS-TEH on SHRs blood pressure was pronounced (Table 4 and Figure 5). Compared with the administration of only TEH hydrogel, the systolic and diastolic blood pressures of SHRs decreased significantly when administered IDDS-TEH, from 2 h to 12 h. Even after removing the electroosmotic hydrogel, the hypotensive effect was maintained for a long duration. In contrast, under normal conditions, the TEH hydrogel cannot deliver drugs into the blood through the skin, and blood pressure fluctuates within a narrow range.

**Table 4.** Effect of IDDS-TEH on blood pressure of SHRs (*n* = 5).

Time (h)	Control group (mmHg)		Current group (mmHg)	
	SBP	DBP	SBP	DBP
0	177.6 ± 4.8	134.8 ± 11.2	181.0 ± 4.3	143.4 ± 12.8
2	159.8 ± 17.9	120.4 ± 16.5	133.4 ± 3.4*	103.4 ± 6.9
4	166.2 ± 9.2	128.8 ± 8.8	129.0 ± 6.4**	101.8 ± 5.5**
6	162.6 ± 1.7	128.4 ± 5.0	140.6 ± 12.0**	107.4 ± 7.0**
8	179.2 ± 3.8	129.0 ± 8.0	159.2 ± 7.0**	117.2 ± 4.1*
24	183.0 ± 5.4	126.0 ± 7.8	187.4 ± 1.8	124.8 ± 5.6
32	184.8 ± 6.9	135.0 ± 8.3	185.6 ± 4.7	131.4 ± 7.0

* indicates a significant difference (*p* < 0.05).

** indicates a significant difference (*p* < 0.01).

## Discussion

In the present study, we provided detailed data regarding the application of our newly developed iontophoresis transdermal delivery system for TEH in guinea pig skin and an SHR model. We measured the current intensity produced from the transdermal iontophoresis delivery system when applied to the guinea pig skin. The *J_ss_* significantly increased following the increase in the current intensity, ranging from 0.10 mA·cm^−2^ to 0.49 mA·cm^−2^. The results indicated that the electrical performance of the iontophoresis transdermal delivery system exhibited potential therapeutic application value. Therefore, the critical factors, including current intensity, drug concentration, pH of the stored drug and the hydrogel, the NaCl concentration, and the chemical penetration enhancer were explored.

First, the concentration of a drug is the most vital factor influencing the transdermal iontophoresis delivery process, which has been investigated in the delivery of several drugs (Abruzzo et al., 2019; Kazemi et al., 2019; Zhang et al., 2019). Generally, the flow increased with increased drug concentrations, such as with metoprolol, butyrate, and diclofenac sodium, among others. However, we found that increased permeation flow flux of drugs did not increase with the TEH concentration when this concentration exceeded a specific level. The skin tissue’s drug channels possibly limit drug permeation; therefore, an excess amount of the drug could not penetrate the skin (Cahusac & Senok, 2020).

Second, ion competition between hydrogen ions and drug ions that carry the current influenced the drug’s penetration (Cheng et al., 2020). When the pH, the concentration of hydrogen ions ([H^+^]), increased from 4.81 to 8.10, a significant increase in *Q* and decreased Flux *J_ss_* was observed. At pH 4.81, TEH iontophoresis predominates compared with other pH values. The highly mobile cation can compete effectively with the positively charged drug to carry current across the skin (Lapteva et al., 2020). However, the donor’s peracid or peralkaline environment will damage the skin barrier, especially when the pH is more than 11 or less than 4, which could irreversibly damage the skin (Işık et al., 2021). This finding illustrates that electro repulsion is an essential mechanism in the iontophoretic delivery of terazosin. In addition, other ions move to the cathode or anode, respectively. Furthermore, Na^+^, Cl^−^, H^+^, and OH^−^ have smaller molecular weights and larger ion mobilities than drug ions, which affect delivery efficiency (Cázares-Delgadillo et al., 2010; Telò et al., 2016). As the concentration of Na^+^ decreased, the amount of drug permeation (*Q*) was reduced. Interestingly, the Flux *J_ss_* at a NaCl concentration of 0.1% was equal to that at pH 4.81, where H^+^ was at its highest concentration. This finding suggests that although Na^+^ is less mobile than H^+^, Na^+^ is also a strong competitor for charge transfer. Previous studies on other transdermal iontophoresis drug delivery systems have also demonstrated the influence of ion competition, such as 5-OH-DPAT, vancomycin, and midazolam (Nugroho et al., 2005; Mohammed et al., 2016; Djabri et al., 2019).

Third, a further experiment was performed to examine whether iontophoresis might provide a safe and effective approach to deliver terazosin to blood *in vivo*. Therefore, SD rats were used as animal models. Terazosin hydrochloride is hydrophilic, and its molecular weight is approximately 500 Da. Generally, it is difficult to pass through the cuticle due to strong fat solubility through passive diffusion. The results showed that no drug permeated the skin under normal conditions. The AUC_(0–_*_t_*_)_ and *C_max_* of TEH through iontophoresis were significantly increased. Moreover, the AUC_(0–_*_t_*_)_ and *C_max_* between the 0.09 and 0.17 mA·cm^−2^ current were significantly different. The larger the current intensity, the larger the AUC_(0–_*_t_*_)_ and *C_max_*. Lastly, this study investigated whether iontophoretic delivery of TEH could be a viable treatment option for hypertensive patients. The iontophoretic delivery of terazosin was applied in SHRs model. After 2 h of administration, the SBP and DBP of SHRs decreased to approximately 80% of that before administration, which indicated the feasibility of transdermal iontophoretic delivery of terazosin. To the best of our knowledge, there are no previously published data concerning iontophoresis in SHR. Further clinical studies are essential to confirm the validity of the approach described here.

## Conclusions

In this study, we aimed to design a system capable of transdermally delivering terazosin hydrochloride, and the characteristics of this system were identified. Transdermal flux was mainly affected by current intensity, pH, drug concentration, and the type of chemical penetration enhancer used. In *vivo* experiments also confirmed the iontophoretic blood concentration efficacy as well as the antihypertensive effect. Based on these findings, further clinical studies should be conducted to validate the feasibility of delivering terazosin at therapeutic doses to hypertensive or benign prostatic hyperplasia patients.

## Data Availability

The data that support the findings of this study are available from the corresponding author (Wenyan Gao), upon reasonable request.
